# 2594. Rate of Methicillin-Resistant *Staphylococcus aureus* Infection Among Patients Hospitalized for Community-Acquired Pneumonia

**DOI:** 10.1093/ofid/ofad500.2209

**Published:** 2023-11-27

**Authors:** Rachel M Poole, Helen Lee, Steven Park

**Affiliations:** UCI Health, Orange, California; UCI Health, Orange, California; University of California, Irvine, Irvine, California

## Abstract

**Background:**

National clinical practice guidelines for community-acquired pneumonia (CAP) suggest empiric treatment for methicillin-resistant *Staphylococcus aureus* (MRSA) among hospitalized patients with risk factors including prior respiratory isolation of MRSA, recent hospitalization with receipt of IV antibiotics within 90 days, or based on a locally validated model. This study aims to assess the rate of MRSA infection among patients hospitalized with CAP to aid clinical decision-making regarding empiric treatment.

**Methods:**

This is a retrospective, observational, population-based, descriptive study at an academic medical center. Patients will be included in the study if they were admitted between 1/01/2021 to 3/18/2023, are at least 18 years old, present with bacterial pneumonia, have a respiratory or blood culture obtained within 48 hours of admission, and complete an MRSA nasal screen, stratified by admission to an ICU vs. non-ICU ward. The primary outcome will be the rate of MRSA infection in patients hospitalized for CAP. The secondary outcome is to determine the negative predictive value (NPV) of MRSA nasal screening. Subgroup analyses will be performed for patients with respiratory MRSA isolation within 1 year of admission, mechanical ventilation within 24 hours of admission, and IV antibiotic use within 90 days of admission.

**Results:**

A total of 1,053 patients met inclusion criteria for the study. The overall rate of MRSA infection was 3.3% (16/490) for ICU patients and 1.1% (6/563) for non-ICU patients. In patients with prior respiratory MRSA isolation, prevalence was 0% (0/7) and 28.6% (2/7) for the ICU and non-ICU groups, respectively. Among mechanically ventilated patients, prevalence was 2% (9/457). For prior IV antibiotic recipients, prevalence was 2.8% (3/109) and 0.5% (1/189) for the ICU and non-ICU groups, respectively. The combined NPV of MRSA nasal screening was 99%.

**Conclusion:**

Overall prevalence of MRSA infection was low among patients hospitalized for CAP, without excluding patients based on underlying comorbid conditions or admission from a skilled nursing facility. MRSA nasal screening was associated with an overall high NPV. Empiric use of vancomycin is unlikely to be warranted for most patients admitted for CAP at our institution.

**Disclosures:**

**All Authors**: No reported disclosures

